# Classification Matters: Divergent Estimates of Dementia Risk Factors in the Health and Retirement Study

**DOI:** 10.1093/geroni/igaf122.3559

**Published:** 2025-12-31

**Authors:** Gina Nam, Junxian Liu, Jacqueline Torres, Eleanor Hayes-Larson

**Affiliations:** USC Leonard Davis School of Gerontology, Los Angeles, California, United States; University of Southern California, Los Angeles, California, United States; University of California, San Francisco, San Francisco, California, United States; University of Southern California, Los Angeles, California, United States

## Abstract

Clinical dementia diagnosis is costly and time-consuming. Therefore, large population studies often use classification algorithms employing various neurocognitive, functional, and health-related measures to identify participants with dementia. We aimed to determine whether choice of classification algorithm affects conclusions regarding dementia risk factors in the nationally representative Health and Retirement Study (HRS). We compared four commonly used classification algorithms (Langa-Weir, Wu, Expert, Hudomiet). For comparability across algorithms, we restricted to HRS participants age 70+ in 2010 and excluded prevalent dementia cases. We followed participants until first classification of dementia, death, loss to follow-up or administrative censoring (2020), calculated dementia incidence rates (IRs), and estimated hazard ratios (HR) from Cox models for dementia risk factors (e.g., sociodemographics, health) adjusted for baseline age, using sampling weights for representativeness. Dementia IRs over follow-up were broadly similar across three algorithms (Langa-Weir, Wu, Hudomiet), ranging from 26.5 (95% CI 24.6-28.4) to 33.8 (31.9-35.8), and higher for Expert (61.7 [59.1-64.7]). HRs for risk factors and incident dementia varied substantially across algorithms; comparing at least high school versus less than high school, HRs ranged from 0.39 [0.35-0.43] (Langa-Weir) to 0.66 [0.56-0.78] (Hudomiet) and comparing Black vs. White participants, HRs ranged from 1.81 [1.54-2.12] (Expert) to 2.69 [2.30-3.15] (Langa-Weir). Because HRS lacks gold standard longitudinal diagnosis and classification algorithms can yield different conclusions, more work is needed to understand optimal choice of algorithm for a given research question.

